# 2-Amino-4-chloro­benzoic acid

**DOI:** 10.1107/S1600536810050166

**Published:** 2010-12-04

**Authors:** Abeer Mohamed Farag, Siang Guan Teoh, Hasnah Osman, Chin Sing Yeap, Hoong-Kun Fun

**Affiliations:** a School of Chemical Sciences, Universiti Sains Malaysia, 11800 USM, Penang, Malaysia; b X-ray Crystallography Unit, School of Physics, Universiti Sains Malaysia, 11800 USM, Penang, Malaysia

## Abstract

The title compound, C_7_H_6_ClNO_2_, is almost planar, with an r.m.s. deviation of 0.040 Å. An intra­molecular N—H⋯O hydrogen bond generates an *S*(6) ring motif. In the crystal, mol­ecules are linked into centrosymmetric dimers by pairs of O—H⋯O hydrogen bonds. These dimers are stacked along [010].

## Related literature

For the pharmacological properties of quinazolinone derivatives, see: Prakash Naik *et al.* (2009 ▶); Bembenek *et al.* (2010 ▶); Miller *et al.* (2010 ▶); Sikorska *et al.* (1998 ▶). For the stability of the temperature controller used in the data collection, see: Cosier & Glazer (1986 ▶). For hydrogen-bond motifs, see: Bernstein *et al.* (1995 ▶).
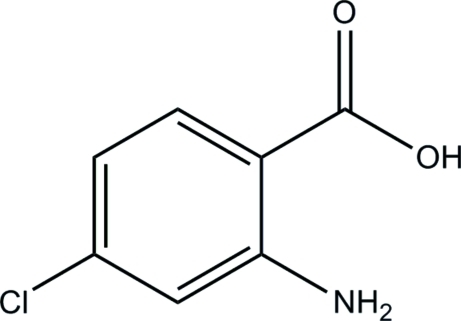


## Experimental

### 

#### Crystal data

C_7_H_6_ClNO_2_
*M* *_r_* = 171.58
Monoclinic, 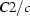

*a* = 15.4667 (10) Å
*b* = 3.7648 (2) Å
*c* = 23.7598 (15) Å
β = 93.015 (3)°
*V* = 1381.59 (14) Å^3^
*Z* = 8
Mo *K*α radiation
μ = 0.49 mm^−1^
*T* = 100 K
0.53 × 0.17 × 0.05 mm

#### Data collection

Bruker APEXII DUO CCD diffractometer
Absorption correction: multi-scan (*SADABS*; Bruker, 2009 ▶) *T*_min_ = 0.780, *T*_max_ = 0.975
34764 measured reflections
3645 independent reflections
3175 reflections with *I* > 2σ(*I*)
*R*_int_ = 0.035

#### Refinement

*R*[*F* ^2^ > 2σ(*F* ^2^)] = 0.030
*wR*(*F* ^2^) = 0.089
*S* = 1.07
3645 reflections
112 parameters
H atoms treated by a mixture of independent and constrained refinement
Δρ_max_ = 0.55 e Å^−3^
Δρ_min_ = −0.21 e Å^−3^

### 

Data collection: *APEX2* (Bruker, 2009 ▶); cell refinement: *SAINT* (Bruker, 2009 ▶); data reduction: *SAINT*; program(s) used to solve structure: *SHELXTL* (Sheldrick, 2008 ▶); program(s) used to refine structure: *SHELXTL*; molecular graphics: *SHELXTL*; software used to prepare material for publication: *SHELXTL* and *PLATON* (Spek, 2009 ▶).

## Supplementary Material

Crystal structure: contains datablocks global, I. DOI: 10.1107/S1600536810050166/hb5757sup1.cif

Structure factors: contains datablocks I. DOI: 10.1107/S1600536810050166/hb5757Isup2.hkl

Additional supplementary materials:  crystallographic information; 3D view; checkCIF report

## Figures and Tables

**Table 1:** Hydrogen-bond geometry (Å, °)

*D*—H⋯*A*	*D*—H	H⋯*A*	*D*⋯*A*	*D*—H⋯*A*
O2—H1*O*2⋯O1^i^	0.853 (16)	1.787 (16)	2.6354 (8)	173.0 (16)
N1—H1*N*1⋯O1	0.851 (15)	2.102 (14)	2.6918 (9)	126.0 (13)

Symmetry code: (i) 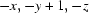.

**Fig. 1.**
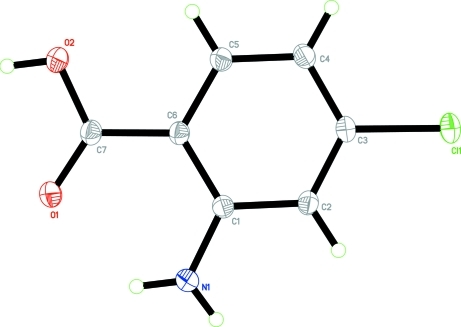
The molecular structure of title compound with 50% probability ellipsoids for non-H atoms.

**Fig. 2.**
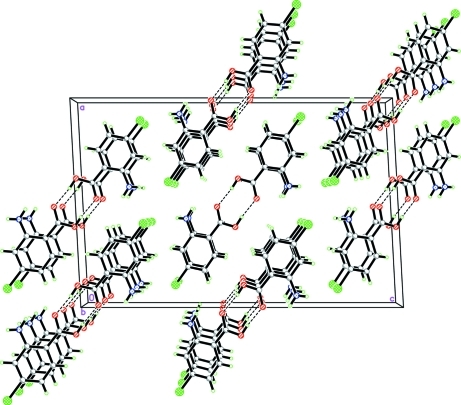
The crystal packing of title compound viewed down b axis, showing the molecules are linked into dimers.

## supplementary crystallographic information

### Comment

Anthranilic acid is required as a starting compound to prepare quinoline derivatives. Quinazolinones are well known as biologically active compounds. Quinazolinones have been studied for their interesting pharmacological properties such as analgesic, antiinflammatory, antibacterial, anticonvulsant, antihypertensive, antimalarial, anticancer activities and as treatment of diabetic complications such as cataracts, nephropathy and neuropathy (Prakash Naik *et al.*, 2009), as well as used as prolyl hydroxylase inhibitors (Bembenek *et al.*, 2010) and antibacterial drugs (Miller *et al.*, 2010). New complexes have been prepared from 2-amino-4-chlorobenzoic acid by Sikorska *et al.*, (1998).

The title compound (Fig. 1) is almost planar with maximum deviation of 0.097 (1) Å at atom O1. An intramolecular N1—H1N1···O1 hydrogen bond generates *S*(6) ring motif (Bernstein *et al.*, 1995). In the crystal, the molecules are linked into centrosymmetric dimers by O2—H1O2···O1 hydrogen bonds and these dimers are stacked down *b* axis (Fig. 2, Table 1).

### Experimental

The attempt to prepare the Schiff base ligand by stirring 2-amino-4-chlorobenzoic acid (1 mol) and salicyldehyde (1 mol) together at 70 °C for 3 h in 10 ml of ethanol was unsuccessful. The resulting orange solution was filtered and orange needles were formed after a few days of slow evaporation of the solvent at room temperature. Unfortunately, the crystals were that of the starting material (2-amino-4-chlorobenzoic acid) with melting point 119 °C.

### Refinement

The O– and N-bound hydrogen atoms were located from difference Fourier map and refined freely. The rest of hydrogen atoms were positioned geometrically [C–H = 0.93 Å] and refined using a riding model [*U*_iso_(H) = 1.2*U*_eq_(C)].

### Crystal data

**Table:**

C_7_H_6_ClNO_2_	*F*(000) = 704
*M**_r_* = 171.58	*D*_x_ = 1.650 Mg m^−^^3^
Monoclinic, *C*2/*c*	Mo *K*α radiation, λ = 0.71073 Å
Hall symbol: -C 2yc	Cell parameters from 9945 reflections
*a* = 15.4667 (10) Å	θ = 2.6–37.5°
*b* = 3.7648 (2) Å	µ = 0.49 mm^−^^1^
*c* = 23.7598 (15) Å	*T* = 100 K
β = 93.015 (3)°	Needle, orange
*V* = 1381.59 (14) Å^3^	0.53 × 0.17 × 0.05 mm
*Z* = 8	

### Data collection

**Table:**

Bruker APEXII DUO CCD diffractometer	3645 independent reflections
Radiation source: fine-focus sealed tube	3175 reflections with *I* > 2σ(*I*)
graphite	*R*_int_ = 0.035
φ and ω scans	θ_max_ = 37.5°, θ_min_ = 1.7°
Absorption correction: multi-scan (*SADABS*; Bruker, 2009)	*h* = −26→26
*T*_min_ = 0.780, *T*_max_ = 0.975	*k* = −6→6
34764 measured reflections	*l* = −38→40

### Refinement

**Table:**

Refinement on *F*^2^	Primary atom site location: structure-invariant direct methods
Least-squares matrix: full	Secondary atom site location: difference Fourier map
*R*[*F*^2^ > 2σ(*F*^2^)] = 0.030	Hydrogen site location: inferred from neighbouring sites
*wR*(*F*^2^) = 0.089	H atoms treated by a mixture of independent and constrained refinement
*S* = 1.07	*w* = 1/[σ^2^(*F*_o_^2^) + (0.0479*P*)^2^ + 0.5195*P*] where *P* = (*F*_o_^2^ + 2*F*_c_^2^)/3
3645 reflections	(Δ/σ)_max_ = 0.001
112 parameters	Δρ_max_ = 0.55 e Å^−^^3^
0 restraints	Δρ_min_ = −0.21 e Å^−^^3^

### Special details

**Table:**

Experimental. The crystal was placed in the cold stream of an Oxford Cryosystems Cobra open-flow nitrogen cryostat (Cosier & Glazer, 1986) operating at 100.0 (1) K.
Geometry. All e.s.d.'s (except the e.s.d. in the dihedral angle between two l.s. planes) are estimated using the full covariance matrix. The cell e.s.d.'s are taken into account individually in the estimation of e.s.d.'s in distances, angles and torsion angles; correlations between e.s.d.'s in cell parameters are only used when they are defined by crystal symmetry. An approximate (isotropic) treatment of cell e.s.d.'s is used for estimating e.s.d.'s involving l.s. planes.
Refinement. Refinement of *F*^2^ against ALL reflections. The weighted *R*-factor *wR* and goodness of fit *S* are based on *F*^2^, conventional *R*-factors *R* are based on *F*, with *F* set to zero for negative *F*^2^. The threshold expression of *F*^2^ > σ(*F*^2^) is used only for calculating *R*-factors(gt) *etc*. and is not relevant to the choice of reflections for refinement. *R*-factors based on *F*^2^ are statistically about twice as large as those based on *F*, and *R*- factors based on ALL data will be even larger.

### Fractional atomic coordinates and isotropic or equivalent isotropic displacement parameters (Å^2^)

**Table:**

	*x*	*y*	*z*	*U*_iso_*/*U*_eq_	
Cl1	0.400495 (11)	0.03406 (5)	0.207389 (8)	0.02000 (6)	
O1	0.01970 (4)	0.31432 (19)	0.06195 (2)	0.02250 (12)	
O2	0.11545 (4)	0.57721 (19)	0.00817 (2)	0.02167 (12)	
N1	0.07764 (4)	0.0546 (2)	0.16254 (3)	0.02152 (13)	
C1	0.15675 (4)	0.1453 (2)	0.14497 (3)	0.01451 (11)	
C2	0.23039 (4)	0.0671 (2)	0.18024 (3)	0.01549 (12)	
H2A	0.2241	−0.0407	0.2150	0.019*	
C3	0.31151 (4)	0.1502 (2)	0.16319 (3)	0.01506 (11)	
C4	0.32545 (4)	0.3164 (2)	0.11206 (3)	0.01691 (12)	
H4A	0.3810	0.3700	0.1015	0.020*	
C5	0.25346 (4)	0.3983 (2)	0.07761 (3)	0.01614 (12)	
H5A	0.2610	0.5113	0.0434	0.019*	
C6	0.16899 (4)	0.31592 (19)	0.09275 (3)	0.01417 (11)	
C7	0.09544 (5)	0.4008 (2)	0.05369 (3)	0.01611 (12)	
H1O2	0.0692 (10)	0.603 (5)	−0.0126 (7)	0.038 (4)*	
H1N1	0.0309 (10)	0.083 (4)	0.1425 (6)	0.034 (4)*	
H2N1	0.0757 (11)	−0.069 (5)	0.1911 (7)	0.042 (4)*	

### Atomic displacement parameters (Å^2^)

**Table:**

	*U*^11^	*U*^22^	*U*^33^	*U*^12^	*U*^13^	*U*^23^
Cl1	0.01555 (8)	0.02243 (10)	0.02140 (9)	0.00327 (6)	−0.00502 (6)	−0.00029 (6)
O1	0.0145 (2)	0.0335 (3)	0.0191 (2)	−0.0014 (2)	−0.00278 (17)	0.0055 (2)
O2	0.0174 (2)	0.0316 (3)	0.0157 (2)	−0.0006 (2)	−0.00240 (18)	0.0067 (2)
N1	0.0141 (2)	0.0308 (4)	0.0196 (3)	−0.0016 (2)	0.0007 (2)	0.0075 (3)
C1	0.0134 (2)	0.0150 (3)	0.0150 (2)	0.0001 (2)	−0.00005 (19)	0.0001 (2)
C2	0.0147 (3)	0.0169 (3)	0.0146 (2)	0.0013 (2)	−0.0012 (2)	0.0011 (2)
C3	0.0136 (2)	0.0155 (3)	0.0158 (2)	0.0016 (2)	−0.00237 (19)	−0.0019 (2)
C4	0.0134 (2)	0.0203 (3)	0.0169 (3)	−0.0005 (2)	−0.0001 (2)	−0.0009 (2)
C5	0.0152 (3)	0.0187 (3)	0.0144 (2)	−0.0007 (2)	0.0000 (2)	−0.0002 (2)
C6	0.0139 (2)	0.0154 (3)	0.0130 (2)	0.0005 (2)	−0.00089 (19)	−0.0004 (2)
C7	0.0159 (3)	0.0183 (3)	0.0140 (2)	0.0013 (2)	−0.00138 (19)	−0.0003 (2)

### Geometric parameters (Å, °)

**Table:**

Cl1—C3	1.7425 (7)	C2—C3	1.3746 (10)
O1—C7	1.2415 (9)	C2—H2A	0.9300
O2—C7	1.3197 (9)	C3—C4	1.3933 (10)
O2—H1O2	0.854 (16)	C4—C5	1.3816 (10)
N1—C1	1.3572 (9)	C4—H4A	0.9300
N1—H1N1	0.851 (16)	C5—C6	1.4078 (9)
N1—H2N1	0.826 (17)	C5—H5A	0.9300
C1—C2	1.4093 (10)	C6—C7	1.4651 (10)
C1—C6	1.4185 (9)		
			
C7—O2—H1O2	107.8 (11)	C5—C4—C3	117.38 (6)
C1—N1—H1N1	123.2 (10)	C5—C4—H4A	121.3
C1—N1—H2N1	117.9 (12)	C3—C4—H4A	121.3
H1N1—N1—H2N1	117.7 (15)	C4—C5—C6	121.95 (7)
N1—C1—C2	118.55 (6)	C4—C5—H5A	119.0
N1—C1—C6	123.17 (6)	C6—C5—H5A	119.0
C2—C1—C6	118.28 (6)	C5—C6—C1	119.45 (6)
C3—C2—C1	119.92 (6)	C5—C6—C7	119.34 (6)
C3—C2—H2A	120.0	C1—C6—C7	121.20 (6)
C1—C2—H2A	120.0	O1—C7—O2	121.70 (7)
C2—C3—C4	123.01 (6)	O1—C7—C6	123.38 (7)
C2—C3—Cl1	118.00 (5)	O2—C7—C6	114.92 (6)
C4—C3—Cl1	118.99 (5)		
			
N1—C1—C2—C3	178.90 (7)	N1—C1—C6—C5	−179.53 (8)
C6—C1—C2—C3	−1.19 (11)	C2—C1—C6—C5	0.57 (11)
C1—C2—C3—C4	0.96 (12)	N1—C1—C6—C7	−0.92 (12)
C1—C2—C3—Cl1	−177.87 (6)	C2—C1—C6—C7	179.18 (7)
C2—C3—C4—C5	−0.05 (12)	C5—C6—C7—O1	174.64 (7)
Cl1—C3—C4—C5	178.77 (6)	C1—C6—C7—O1	−3.97 (12)
C3—C4—C5—C6	−0.59 (12)	C5—C6—C7—O2	−5.06 (11)
C4—C5—C6—C1	0.33 (11)	C1—C6—C7—O2	176.34 (7)
C4—C5—C6—C7	−178.31 (7)		

### Hydrogen-bond geometry (Å, °)

**Table:**

*D*—H···*A*	*D*—H	H···*A*	*D*···*A*	*D*—H···*A*
O2—H1O2···O1^i^	0.853 (16)	1.787 (16)	2.6354 (8)	173.0 (16)
N1—H1N1···O1	0.851 (15)	2.102 (14)	2.6918 (9)	126.0 (13)

Symmetry codes: (i) −*x*, −*y*+1, −*z*.
